# A joint time-assignment and expenditure-allocation model: value of leisure and value of time assigned to travel for specific population segments

**DOI:** 10.1007/s11116-019-10022-w

**Published:** 2019-06-25

**Authors:** Reinhard Hössinger, Florian Aschauer, Sergio Jara-Díaz, Simona Jokubauskaite, Basil Schmid, Stefanie Peer, Kay W. Axhausen, Regine Gerike

**Affiliations:** 1 Institute for Transport Studies, University of Natural Resources and Life Sciences, Vienna, Austria; 2 Department of Civil Engineering, University of Chile, Santiago, Chile; 3 Institute of Applied Statistics and Computing, University of Natural Resources and Life Sciences, Vienna, Austria; 4 Institute for Transport Planning and Systems, ETH Zurich, Zurich, Switzerland; 5 Institute for Multi-Level Governance and Development, WU Vienna, Vienna, Austria; 6 Integrated Transport Planning and Traffic Engineering, TU Dresden, Dresden, Germany

**Keywords:** Value of leisure, Value of time assigned to travel, Time use, Expenditure allocation, Segmentation

## Abstract

Based on a time-use model with a sound theoretical basis and carefully collected data for Austria, the value of leisure (VoL) for different population segments has been estimated. Through the combination of these results with mode-specific values of travel time savings from a related study based on the same data, the first mode-specific values of time assigned to travel (VTAT) were calculated. Data was collected using a Mobility-Activity-Expenditure Diary, a novel survey format which gathers all activities, expenditures, and travel decisions from the same individuals for 1 week in a diary-based format. The average VoL is 8.17 €/h, which is below the mean wage of 12.14 €/h, indicating that the value of work is, on average, negative. Regarding the reliability of the VoL, we show its sensitivity to the variance of working time in a sample, something that has been ignored in previous studies and could be used to avoid inadequate segmentation. We controlled this effect in the analysis of the heterogeneity of the VoL across the population by estimating the parameters from the total (unsegmented) dataset with single interaction terms. We find that the VTAT is strictly negative for walking, predominantly negative for cycling and car, and predominantly positive for public transport with 0.27 €/h on average. The positive VTAT for public transport is a strong indication for the importance of travel conditions, in turn suggesting that improvements in travel conditions of public transport might be as important as investing in shorter travel times.

## Introduction

Jara-Díaz and Guevara (2003) highlighted that a person who makes a travel decision not only maximizes her utility in this particular choice, but also in the surrounding time-expenditure space. In order to combine both components, they developed a theoretical time-use framework model which can be applied to obtain values for different aspects of time use. A key output is the value of leisure (VoL) which represents the value of the marginal utility of all activities that are assigned more time than the minimum necessary. Following DeSerpa (1973), the authors show that estimating the VoL permits a deeper examination of the value of travel time savings (VTTS) obtained from travel choice models because the VTTS equals the VoL minus the value of time assigned to travel (VTAT). The intuition behind this is that the VTTS summarizes the value of the liberated time (opportunity cost of travel), while the VTAT represents the ‘loss’ when travel time is reduced—which is why it relates to travel conditions. The VoL is, therefore, a key piece of information for the integration of travel decisions into the framework of consumer home production. Furthermore, the VTAT is also important because it represents the direct utility (or disutility) derived from the time spent in the travel activity. The VTAT may differ between modes and according to specific conditions of travel such as comfort, reliability, crowding, or the possibility to use the in-vehicle time productively. There is some indication that the increasing availability of mobile devices enables public transport passengers to use the in-vehicle time more productively, which may yield a higher value of time assigned to public transport (e.g. Litman 2008). In particular, train travel time can be used for many activities (Lyons et al. 2013). Flügel (2014) provided a summary of why public transport travellers may perceive travel as more relaxed than car travellers.

The VTTS is usually obtained from (conditional) indirect utilities estimated using discrete choice models; it represents the total marginal willingness-to-pay for a reduction of travel time in the context of travel choices.^1^ The VTTS has high practical relevance in transport planning because savings in travel time account for the biggest share of user benefits in most cost–benefit analyses (e.g. Jara-Díaz 1990; Wardman and Lyons 2016; Hensher and Wang 2016). Mode choice models are able to estimate the VTTS by travel mode.

Obtaining the VTAT is more difficult: to be computed it requires the VoL and the VTTS. Estimating the VoL requires in turn a large amount of information from each individual, most importantly time assignment patterns, the allocation of expenditures to various commodities, and travel decisions over a period of sufficient length to be considered as the long-term equilibrium of the individual (Jara-Díaz and Rosales-Salas 2015, 2017). As a consequence, only a few attempts have been made so far to estimate the VoL with the aforementioned model framework. Table 1 lists the results obtained with the original model formulation presented by Jara-Díaz and Guevara (2003) and later expanded by Jara-Díaz et al. (2008). They reveal a huge variability of VoL estimates ranging from 0.12 to 123 €/h, and the ratio VoL/w ranges between 0.04 and 6.83. Note, however, that the results from Jara-Díaz and Guevara (2003) were obtained with a limited preliminary version of the model. Also, the results from the Netherlands reported by Jara-Díaz et al. (2016) are rather implausible; this is discussed at the end of “Data preparation” section. If these studies are not considered, the ratio VoL/w moves only from 0.57 to 2.48. Nonetheless, only a small part of this range can be explained by socio-demographic characteristics or structural factors such as survey year or the economic level of the country. Most VoL estimates follow the order of each country’s well-being from the World Values Survey (Frey and Stutzer 2002), but the differences are too large to result from this factor alone. The main part of variability remains unexplained; this raises the question how to estimate the VoL to reflect the time and cost preferences of in a reliable manner.

**Table 1:** Values of leisure (VoL) estimated from microeconomic time-use models), wage rates (w), and ratios between them reported in the literature

Source	Country	Survey year	Segment	VoL (€/h)	w (€/h)	VoL/w
Jara-Díaz and Guevara (2003)	Chile	1991	Medium income	0.12	3.12	0.04
	Chile	1991	High income	0.31	6.90	0.05
Munizaga et al. (2008)	Chile	2001	Total sample	3.07	4.97	0.62
Jara-Díaz et al. (2008)	Chile	1991	Total sample	2.31	3.50	0.66
	Germany	1999	Total sample	11.92	9.95	1.20
	Switzerland	2003	Total sample	23.66	26.94	0.88
Jara-Díaz and Astroza (2013)	Chile	2001	Women	2.25	2.44	0.92
	Chile	2001	Men	1.78	3.11	0.57
Jara-Díaz et al. (2013)	Chile	2001	Men, SCL-East	6.82	9.61	0.71
	Chile	2001	Men, SCL-S.East	2.24	2.68	0.83
	Chile	2001	Men, SCL-West	1.56	2.12	0.74
	Chile	2001	Men, SCL-North	1.68	1.90	0.88
	Chile	2001	Men, SCL-South	1.45	1.90	0.76
	Chile	2001	Women, SCL-East	6.48	5.92	1.09
	Chile	2001	Women, SCL-S.East	2.57	2.12	1.21
	Chile	2001	Women, SCL-West	2.12	1.79	1.19
	Chile	2001	Women, SCL-North	2.24	1.68	1.33
	Chile	2001	Women, SCL-South	2.12	1.68	1.27
Konduri et al. (2011)	USA	2008	Low income	12.70	10.50	1.21
	USA	2008	Medium income	14.31	17.42	0.82
	USA	2008	High income	76.75	30.97	2.48
	USA	2008	Women	24.56	21.52	1.14
	USA	2008	Men	39.94	18.17	2.20
Jara-Díaz et al. (2016)	Netherlands	2012	Full sample	122.80	17.99	6.83

Results in nominal prices and converted to Euros per hour; exchange rates for conversion were gained from three sources

1991–1994: Federal Reserve Economic: https://fred.stlouisfed.org/series/EXUSEC?cid=280

1995–1998: OECD: https://data.oecd.org/conversion/exchange-rates.htm

Since 1999: World Bank: https://data.worldbank.org/indicator/PA.NUS.FCRF

A possible source of unsystematic fluctuations are deficits and gaps in the data. One of the Chilean samples includes a specific population segment (long-distance commuters to downtown Santiago) who completed a 3-day activity diary—expenditures were not reported (Jara-Díaz et al. 2004; Munizaga et al. 2008). Other Chilean data bases were constructed from origin–destination surveys (Jara-Díaz and Guevara 2003; Jara-Díaz et al. 2013).

The German and Swiss data are based on a 6-week travel diary—expenditures were not reported and non-travel activities were inferred from the trip purposes (for Germany: Axhausen et al. 2002; for Switzerland: Löchl et al. 2005). The Dutch results (Jara-Díaz et al. 2016) are based on the LISS panel (Longitudinal Internet Studies for the Social Sciences), which is a retrospective survey of average activity durations and expenditures; trip details such as travel modes are not reported. Finally, the U.S. results are based on a synthetic dataset obtained from a probabilistic merge of participants of a time-use survey and a consumer expenditure survey (Konduri et al. 2011). The dataset has been used to estimate various time-use and expenditure models including the multiple discrete–continuous extreme value model (MDCEV, see Castro et al. 2012). Both time-use and expenditure information is assuredly of high quality, but the probabilistic merge is questionable given that the aim of such models is to estimate trade-offs between time-use and expenditures at the individual level. This calls for a simultaneous survey of both components. To our best knowledge, no dataset exists so far with information on time assignment (including travel details) and expenditures, which has been collected from the same individuals at the same time in a diary-based format.

The unavailability of appropriate data to estimate all values of time (leisure, work, VTTS, VTAT) is the starting point of the current study. We are contributing to this aspect of research by using a novel comprehensive dataset for model estimation, which was obtained from a Mobility-Activity-Expenditure Diary (MAED). This dataset includes information on activity assignment, expenditure allocation, and travel decisions over 1 week (considered as the whole work-leisure cycle) in a diary-based survey format, which has been proven reliable and valid in time-use surveys, consumer expenditure surveys, and travel surveys. Using these data, many objectives can be achieved:

(i) Estimation of the VoL for different population segments with three alternative approaches:
  1. A priori segmentation: subdivide the dataset into segments and estimate separate models for each one;
  2. Ex-post segmentation: estimate a global model and calculate the VoL from segmented result datasets;
  3. Ex-post segmentation with interaction terms: like (i2) but with moderator variables and interaction terms.
    Previous research has used a priori segmentation which estimates all parameters group-specific, for example, the results of Jara-Díaz and Astroza (2013) and Jara-Díaz et al. (2013) included in Table 1. Ex-post segmentation is more efficient, as it does not require dividing the sample into small groups. It would be a methodological advance if ex-post segmentation (without or with interaction terms) proves to be suitable.
(ii) Estimation of the value of time assigned to travel (VTAT) with two innovations: (ii1) mode-specific estimation, which yields a separate VTAT for each travel mode, and (ii2) estimation based on the complete model framework introduced by Jara-Díaz and Guevara (2003), including travel choices, activity assignment, and (for the first time) expenditure allocation. The VTAT requires the VoL and the VTTS to be calculated.^2^

## Model

Our model is based on the formulation created by Jara-Díaz et al. (2008). We used this model as a benchmark because it has been applied to four countries (Chile, Germany, Switzerland, USA) as well as to many segments within two of those countries (Chile and USA). This allows our estimations to have a basis for comparison (see Table 1). Note that the MAED data that are used in this paper do not provide a one-to-one mapping between activities and goods, and accounting for these relations would require assumptions which are not needed in the basic 2008 model.^3^ Here we explicitly recognize that activities have a cost through the market goods bought but we do not attempt to find the proportions by which expenses are allocated to individual activities.

Our utility function *U* is shown in Eq. (1). It is the log-linear version of a Cobb–Douglas function including three terms which relate to the utility gained from time assigned to work, time assigned to leisure, and expenses assigned to freely consumed goods. The logarithms enforce diminishing marginal utility as the consumption level of a particular alternative increases (i.e., satiation). This assumption yields a multiple discreteness model—that is, the choice of multiple alternatives can occur simultaneously (see Bhat 2005, 2008).^4^

1 $$ U = \theta_{w} \log \left( {T_{w} } \right) + \mathop \sum \limits_{i = 1}^{n} \theta_{i} \log \left( {T_{i} } \right) + \mathop \sum \limits_{j = 1}^{m} \varphi_{j} \log \left( {E_{j} } \right) $$

The utility-generating resources (time *T* and expenses *E*) are subject to the following constraints:

2 $$ \tau - T_{w} - \sum\limits_{i = 1}^{n} {T_{i} = 0 \left( \mu \right)} \quad {\text{time}}\;{\text{constraint}} $$

3 $$ wT_{w} + I - \sum\limits_{j = 1}^{m} {E_{j} \ge 0 \left( \lambda \right)} \quad {\text{budget}}\;{\text{constraint}} $$

4 $$ T_{i} - T_{i}^{Min} \ge 0, \;\forall i \in A^{r} \left( {\kappa_{i} } \right)\quad  {\text{technical}}\;{\text{constraint}}\;{\text{on}}\;{\text{committed}}\;{\text{activities}} $$

5 $$ E_{j} - E_{j}^{Min} \ge 0,\, \forall j \in G^{r} \left( {\eta_{j} } \right)\quad {\text{technical}}\;{\text{constraint}}\;{\text{on}}\;{\text{committed}}\;{\text{goods}} $$

$\theta_{w}$ is the baseline utility of assigning time to work; *T*_*w*_ the amount of time assigned to work; $\theta_{i}$ and *T*_*i*_ the baseline utility and amount of time assigned to activity *i*; $\varphi_{j}$ and *E*_*j*_ the baseline utility and amount of expenses assigned to good *j*; $\tau$ the total time constraint; *w* the wage rate; *I* fixed income from other sources but work; *μ* and *λ* are Lagrange multipliers representing the marginal utility of increasing available time and increasing available income; *κ*_*i*_ the Lagrange multiplier representing the marginal utility of reducing the minimum time constraint of restricted activity *i *ϵ *A*^*r*^; and *η*_*i*_ the Lagrange multiplier representing the marginal utility of reducing the minimum expenditure constraint of restricted good *j *ϵ *G*^*r*^. Committed activities and goods are those which are necessary for personal and household maintenance such as travel, cleaning the house, rental cost, etc. They are limited at the bottom by technical constraints (i.e., people would like to assign less time and money but cannot because of the technical constraints). The amount of time and expenses assigned to these activities and goods is given externally. It is inferred from the observations and included in the equations as *T*_*C*_ and *E*_*c*_ (see below).

Furthermore, we assume that each individual assigns non-zero amounts of time and money to each unconstrained activity and consumed good because the logarithms in Eq. (1) do not allow zeros. This is reasonable as we are dealing with an aggregated view of activities and expenses assigned to a work-leisure cycle (only one category of work, leisure, and expenses during a whole week), which prevents the presence of zero assignments.^5^ The original form of the Jara-Díaz et al. (2008) model was stated in terms of goods consumption *X*_*j*_, which is represented here by expenses assigned to goods in monetary terms $E_{j} = P_{j} X_{j}$, where *P*_*j*_ is the unit price of good *j*. This will be shown to be equivalent to the original model in Eq. (15) below.

Following Jara-Díaz et al. (2008), we obtain the first order conditions to find the optimal allocation of activities and expenditures. They yield a solution for *T*_*w*_, *T*_*i*_, and *E*_*j*_, which can be used to calculate $\mu$ and $\lambda$, and consequently the VoL and VTAT. The first order conditions are:

6 $$ \frac{{\theta_{w} }}{{T_{w} }} + \lambda w - \mu = 0 $$

7 $$ \frac{{\theta_{i} }}{{T_{i} }} - \mu = 0,\; \forall i \in A^{f} $$

8 $$ \frac{{\varphi_{j} }}{{E_{j} }} - \lambda = 0,\; \forall j \in G^{f} $$

where *A*^*f*^ and *G*^*f*^ denote the set of freely chosen activities and freely consumed goods, respectively. Equation (8) is derived from the budget constraint in Eq. (3) which is always binding when maximizing U, such that $\lambda$ is always positive.

Calculate $\mu$ and $\lambda$ from first order conditions:

9 $$ \mu = \frac{\partial U}{{\partial T_{i} }} = \frac{\varTheta }{{\left( {\tau - T_{w} - T_{c} } \right)}} $$

10 $$ \lambda = \frac{\partial U}{{\partial E_{j} }} = \frac{\varPhi }{{\left( {wT_{w} - E_{c} } \right)}} $$

The parameters *Θ* and *Φ* correspond to the sum of individual time coefficients $\theta_{i} (\varTheta = \sum\nolimits_{{i \in A^{f} }} {\theta_{i} } )$ and individual expenditure coefficients $\varphi_{j} (\varPhi = \sum\nolimits_{{i \in G^{f} }} {\varphi_{j} } )$.

Re-write (6) to (11) and insert (9) and (10) into (11):

11 $$ T_{w} \left( {\lambda w - \mu } \right) + \theta_{w} = 0 $$

12 $$ T_{w} \left[ {\frac{\varPhi w}{{\left( {wT_{w} - E_{c} } \right)}} {-} \frac{\varTheta }{{\left( {\tau - T_{w} - T_{c} } \right)}} } \right] + \theta_{w} = 0 $$

Solve the quadratic Equation in (10) to obtain the optimal working time $T_{w}^{*}$:

13 $$ T_{w}^{*} = \frac{{\left( {\varPhi + \theta_{w}  } \right)\left( {\tau - T_{c} } \right) + \frac{{E_{c} }}{w}\left( {\varTheta + \theta_{w} } \right) \pm \sqrt {\left[ {\frac{{E_{c} }}{w}\left( {\varTheta + \theta_{w} } \right) + \left( {\tau - T_{c} } \right)\left( {\varPhi + \theta_{w}  } \right)} \right]^{2} - 4\frac{{E_{c} }}{w}\left( {\tau - T_{c} } \right)\theta_{w} \left( {\varTheta + \varPhi + \theta_{w} } \right)} }}{{2\left( {\varTheta + \varPhi + \theta_{w} } \right)}} $$

Insert (9) into (7) with $T_{w}^{*}$ to obtain $T_{i}^{*}$:

14 $$ T_{i}^{*} = \frac{{\theta_{i} }}{\varTheta }\left( {\tau - T_{w}^{*} - T_{c} } \right) $$

Insert (10) into (8) with $T_{w}^{*}$ to obtain $E_{j}^{*}$^6^:

15 $$ E_{j}^{*} = \frac{{\varphi_{j} }}{\varPhi }\left( {wT_{w}^{*} - E_{c} } \right) $$

Please note that there is a difference between our equation system and the one proposed by Jara-Díaz et al. (2008). They normalised their parameters to $2(\varTheta + \varPhi + \theta_{w} )$. This yields a simplified equations system (20)–(22), which was used to estimate normalised parameters $\alpha$ and $\beta$. We normalised our parameters by setting *Θ* to one. This enables us to estimate the original parameters directly from Eqs. (13) to (15).^7^ The VoL and VTAW are then obtained from the estimated parameters by inserting (9) and (10) into (6):

16 $$ VoL = \frac{{\partial U/\partial T_{i} }}{{\partial U/\partial E_{j} }} = \frac{\mu }{\lambda } = \frac{{\varTheta \left( {wT_{w} - E_{c} } \right)}}{{\varPhi \left( {\tau - T_{w} - T_{c} } \right)}} $$

17 $$ VTAW = \frac{{\partial U/\partial T_{w} }}{{\partial U/\partial E_{j} }} = \frac{\mu }{\lambda } - w = VoL - w $$

## Data

A relevant new aspect of the paper at hand is the estimation of time-use models from a dataset in which activities and expenditures are obtained simultaneously from the same individuals in a diary-based survey. The underlying dataset is discussed in detail in papers by Aschauer et al. (2018, 2019). The sample provides information about all activities, expenditures, and travel decisions over a period of 1 week. It is based on a novel survey design, the Mobility-Activity-Expenditure Diary (MAED), and a survey conducted in spring and autumn 2015. It is a self-administered mail-back survey with a 1-week reporting period, including questions concerning trips, activities, and expenditures for each diary day. The trip section resembles the traditional household travel survey format based on the New KONTIV design^8^ (Brög et al. 2009; Socialdata 2009), but the trip purpose section is more comprehensive. It resembles a time-use diary but with predefined activity types instead of open text fields. Each activity type is reported in a separate row along with the start time, end time, and possible expenditures, which are specified by means of their amount and type. The classification of expenses follows the UN standard Classification of Individual Consumption According to Purpose (COICOP; see UN 2018).

### Sampling

**Fig. 1:**
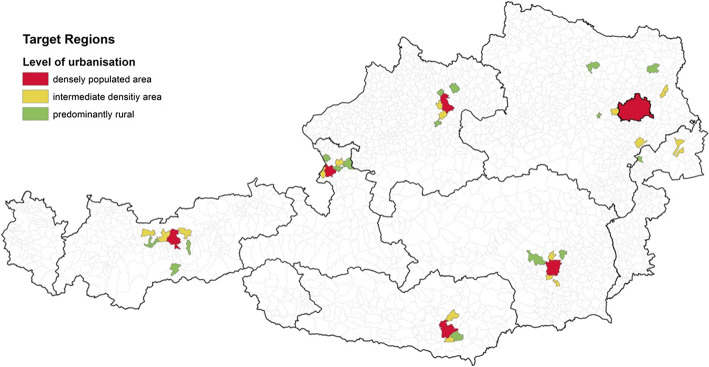
Survey locations in Austria

The sample was based on a random selection of Austrian households for 18 pre-defined strata defined by region and level of urbanisation as shown in Fig. 1. Only employed persons were selected for participation because a wage rate is required for model estimation. The survey procedure followed the household travel survey tradition with some modifications resulting from the necessity of screening for employed persons and the high respondent burden as explained in detail in Aschauer et al. (2018, 2019), where the MAED data are presented and compared to the latest Austrian travel survey, time-use survey, and expenditure survey.

### Data preparation

Aside from usual plausibility checks, two additional adjustments were necessary in order to reduce the incidental variation in the diary data and to better reflect the long-term equilibrium of the individuals.

#### Adjustment of activity durations

A key problem with respect to activities is the working time reported in the diary. It can deviate from the usual amount due to incidental events during the reporting week, such as workload peaks, bank holidays, sickness, training courses, etc. The result is an unsystematic variation of the reported working time which causes unrealistic balances of income and expenditures, because the working time (along with the wage) determines the implied income in the time-use model [see Eq. (3)]. We addressed this problem by asking for the regular hours worked (according to the contract) and the usual hours of overtime in the personal questionnaire which accompanied the diary. For data analysis, we replaced the reported working time in the diary for all respondents with the ‘effective working time’, which is the sum of the regular working time and the usual hours of overtime. The durations of non-work activities were adjusted accordingly to satisfy the time constraint. We assume an asymmetric adjustment pattern in the sense that an incidental increase of working time (beyond the usual level) causes different re-arrangement patterns than an incidental reduction (below the usual level). For this purpose, we estimated two separate models which were used for the adjustment of activities of two different groups:

1. Persons who worked more than usual in the reporting week: reduce the working time to the ‘usual effective working time’ and increase non-work activities accordingly in order to meet the time constraint;
2. Those who worked less than usual: increase the working time and reduce non-work activities accordingly.

#### Adjustment of expenditures

Linked to the reporting of expenditures is the large variability of purchase rhythms of goods and services. In line with conventional expenditure surveys, expenditure information was collected in two sections of the questionnaire: frequently purchased items were reported in the diary, whereas long-term expenses were reported in the household section. This requires a procedure of combining both sources in a manner that avoids double-counting through expenses that occur in both sections. Aschauer et al. (2019) describe the procedures that have been tested and applied in this context. The collection of expenditure data at two levels (personal and household) induces the need of some rule to allocate the expenses to those individuals who generate income (i.e. earners). The default MAED dataset is based on ‘proportional expenses’ according to the labour income of the household members. In order to run a sensitivity analysis we generated an alternative dataset based on ‘equal expenses’ for freely chosen goods. It assumes transfer payments within the household such that all earners have equal amounts available for freely chosen expenses. In “Value of leisure (VoL) and value of time assigned to work (VTAW)” section (Table 3) we provide and discuss the results of both datasets.

A second issue associated with the expenses is the large variation of short-term expenses in the diary. A randomly selected week can deviate from the long-term equilibrium for two reasons: exceptionally large purchases (one-time-big-ticket items, e.g. a new car) and implausible zero spending on essential goods such as food or travel. Reported zeros may be reduced by a longer observation period and face-to-face support of participants, as is usual for conventional expenditure surveys, but this has not been done in the MAED because of the unacceptable response burden given that the participants also reported their trips and activities. We employed a model-based smoothing of expenditures with the intention to reduce the large incidental variation caused by the aforementioned problems but to retain the individual variability as much as possible. The applied procedure consisted of three steps:

1. Predict the total expenditures as the difference between reported income and estimated savings; the monthly savings are estimated by a linear model using personal and household characteristics as predictors.
2. Predict the expenditure shares by category with a multinomial logit model, again using personal and household characteristics as predictors.
3. Replace the reported total expenditures with the predicted total expenditures (Step 1) and fix the balance by adjusting individual expenditure categories using the predicted expenditure shares (Step 2) as a benchmark.

This procedure ensured that the reported expenditures were carefully adjusted (1) only to the necessary extent in order to fix the balance between income, savings, and expenditures, and (2) towards a benchmark which is already adapted to individual characteristics by the multinomial logit model.

All models used for the adjustment are provided in the “Appendix” section (Tables 7, 8, 9 and Fig. 10). The models comprise many predictors including insignificant ones. This is in accordance with the purpose of the adjustment: we did not attempt to obtain the most parsimonious model (as usual for prediction models) but a rich model that reproduces the highest possible share of individual variability. Table 2 shows the pairwise correlations between reported and adjusted amounts. The average correlation is 0.94 for activities and 0.85 for expenditures. The lower correlation of expenditures results from their larger variability: the coefficient of variation of reported expenditures is more than two times higher than that of activities. The generally high level of correlations (also for expenses) indicates that the major portion of reported variability could be maintained with the adjusted data.

**Table 2:** Pairwise correlations between reported and adjusted activity durations and expenditures

Activity category	Correlation	Expenditure category	Correlation
Travel	0.982	Housing	0.883
Sleep	0.946	Food	0.874
Eating	0.984	Accommodation	0.871
Education	0.995	Clothes	0.877
Personal	0.983	Furniture	0.899
Domestic	0.985	Health	0.901
Shopping	0.999	Mobility	0.895
Leisure	0.925	Electronics	0.859
Work	0.648	Leisure	0.869
		Education	0.898
		Service	0.861
		Finances	0.892
		Insurance	0.889
		Others	0.825
		Savings	0.524

The MAED data rectify some limitations of existing datasets that include time-use and expenditures and have been used in previous estimations of time-use models. Our data were obtained directly from the same individuals in a diary format. This is an advantage over retrospective data collections (such as in the Dutch LISS panel) because retrospective questions can lead to biased mean values (Browning and Gørtz 2006). Moreover, obtaining activities and expenditures simultaneously from the same individuals should be preferred over imputing expenditures from external sources as done by Konduri et al. (2011), because a time-use model is mainly about individuals’ trade-offs between time-use, income, and expenditures. Note, however, that the probabilistic merge of participants of a time-use survey with those of a consumer expenditure survey has one possible strength: consumer expenditure surveys collect the expenses usually with more effort over a longer time period than combined surveys (such as the MAED survey) which will most likely result in more accurate data (while also requiring some adjustment and averaging). Finally, we believe that any data obtained from a diary-based survey should undergo an adjustment to fix the individual balances between work time, income, and expenses because data used for a time-use model should represent the long-term equilibrium on individual levels and not simply as an average across the sample.

### Data description

**Fig. 2:**
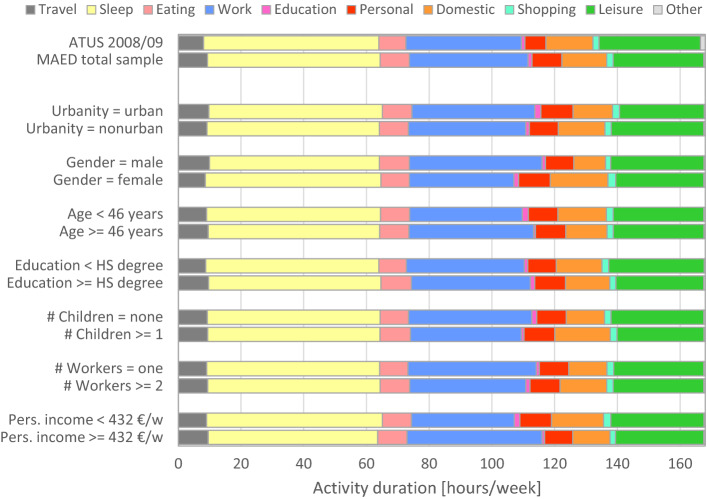
Average duration by activity category and population segments (ATUS = Austrian Time Use Survey)

Figure 2 shows the average activity duration per activity category during the reporting week. The top two bars compare the MAED with the latest Austrian time-use survey (ATUS); we included only employed persons of the ATUS to be consistent with the MAED. Apart from minor deviations, the MAED results fit the time distribution of the ATUS very well. The largest difference is a shortfall of leisure activities in favour of travel and personal activities. Both shifts are probably caused by methodological differences. In the MAED we took great care to record all trips, whereas time-use surveys are well known for under-reporting trips (Gerike et al. 2015 as well as Aschauer et al. 2018). The shift from leisure to personal activities in the MAED is very likely caused by different coding schemes. MAED participants coded the activity types themselves (such as personal or leisure) based on our instructions; one instruction was that ‘leisure’ should be coded if the activity was performed voluntarily. ATUS participants stated the specific kind of activity in open text fields (such as reading or playing with the children). The abstract activity types were inferred from these statements during data processing, but, there is a broad overlap between personal and leisure; many activities that were inferred as ‘leisure’ from the ATUS statements could be perceived as duty by the participants—in particular, social activities such as going to church or visiting a hospital patient. MAED participants would have coded ‘personal’ in this case.

The remaining bars in Fig. 2 show the average activity durations across different population segments in the MAED sample; they reveal only small differences. If there is a horizontal shift across the segments, it is in most cases a trade-off between paid work and unpaid (domestic) work. This shift is most pronounced in the difference between men and women. The particularly high substitution rate between paid work and domestic work is reflected by the largest negative correlation (− 0.93) among all pairwise correlations between activity categories.

**Fig. 3:**
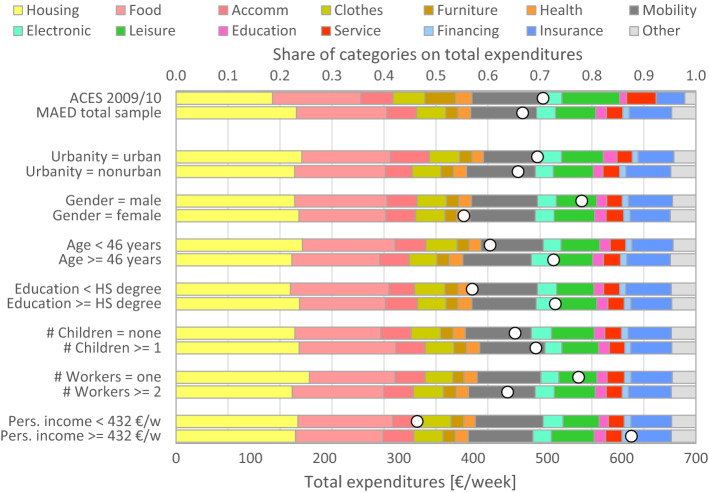
Personal expenses by population segments (ACES = Austrian Consumer Expenditure Survey); the white dots show the total expenses with respect to the lower axis; the coloured bars show the average shares of expenditures by category with respect to the upper axis. (Color figure online)

Figure 3 shows the total weekly expenses (white dots) and shares of expenditures by category (coloured bars). The total expenses differ greatly between the segments, most of all between low and high-income (as expected) with a ratio of 1.9; but other segments reveal large differences as well: men, older persons, persons with higher education, and persons living in single-worker households spend more money than those in the complementary segments. The two bars at the top compare the MAED sample with the latest Austrian consumer expenditure survey (ACES), including only employed persons to be consistent with the MAED. The differences are larger than those between MAED and ATUS (see Fig. 2), possibly reflecting the difficulties of surveying expenditures (see “Data preparation” section). The largest deviation (4.6%) refers to the share of housing; it has a specific reason: the original ACES includes rental equivalents (instead of reported expenses) of owner-occupied housing. The MAED data include, in contrast, reported mortgage repayments and operating costs, which are not comparable to (on average lower than) the rental equivalents. Since we found no way to match both procedures, we removed the rental equivalents in the ACES, which explains the lower share.

The remaining bars in Fig. 3 show the average shares of expenditures across different population segments in the MAED sample. The variability of the shares across the segments is much smaller than that of total expenses, which means that people with higher income spend more money on all kinds of commodities: they live in more expensive houses, eat more expensive food, wear more expensive clothes, etc. From this pattern we can conclude that the Cobb–Douglas function holds for the expenditures in the sense that “having chosen the ultimately satisfying budget shares at any given set of relative prices, the superlatively wealthy continue to allocate additional income in the same proportions” (Powell et al. 2002).

## Results

### Value of leisure (VoL) and value of time assigned to work (VTAW)

The model estimation requires to classify the reported activity and expenditure categories into the model variables. The model defines three types of decision variables: (1) duration of paid work [*T*_*W*_ in Eq. (6)], (2) duration of freely chosen activities [*A*^*f*^ in Eq. (7)] to which people assign more time than the technical minimum, and (3) expenses on freely consumed goods [*G*^*f*^ in Eq. (8)] which people consume more than the technical minimum. These three types of variables allow for a closed-form solution; the resulting equation system (13)–(15) can be used to estimate the utility parameters and to calculate the marginal values of leisure and work by inserting the estimates in Eqs. (16) and (17). Furthermore, the model defines two types of exogenous variables referred to as committed activities [*T*_*c*_ in Eq. (9)] and committed expenses [*E*_*c*_ in Eq. (10)]. We assume that the consumption levels of these committed variables are externally determined by technical constraints, which require a certain minimum (Jara-Díaz 2003) and leave no choice to the consumers but to stick to this minimum.

Table 3 shows how the reported activity and expenditure categories were assigned to the model variables. The allocation is critical because it is arbitrary (cannot be deducted from the data) but affects the result. Our definition of committed activities (*T*_*C*_) follows the classification of Jara-Díaz et al. (2013), who identified six types: household chores, personal care, assisting friends and family (‘other’ in the MAED sample), administrative chores and family finances, commuting, and education. The only exception is ‘sleep’, which Jara-Díaz et al. classified as free activity, whereas we belief that most people try to stick to the minimum. Personal care and household chores are typically classified as ‘committed’ because of their maintenance-oriented nature (Bittman and Wajcman 2000; Robinson and Godbey 2010). These activities are driven by a physical need, but, in most cases, people do not want to pay more attention than necessary. Gronau and Hamermesh (2006) classified these activities as ‘goods intensive’, that is, individuals particularly care about the amount of goods assigned to them. Ahn et al. (2004) also found that people try to save money in maintenance activities. Travel time might, in principle, be considered as an endogenous variable, which is related to activity destinations and also to the overall framework of time and budget assignment as shown by Jara-Díaz and Guerra (2003). However, in this paper we follow the approach by Jara-Díaz et al. (2008) which, in essence, states that *ceteris paribus* individuals would be willing to reduce travel time but cannot due to the characteristics of the transport system (transit design, road network, etc.).

**Table 3:** Classification of observed activities and expenditures into model variables

Observed activity category	Model variable	Observed expenditure Category	Model variable
Work	*T*_*W*_	Leisure	*E*_1_
Leisure	*T*_1_	Accommodation	*E*_1_
Eating	*T*_2_	Electronic	*E*_1_
Shopping	*T*_2_	Clothes	*E*_2_
Sleep	*T*_*C*_	Housing	*E*_*C*_
Personal	*T*_*C*_	Food	*E*_*C*_
Domestic	*T*_*C*_	Furniture	*E*_*C*_
Education	*T*_*C*_	Health	*E*_*C*_
Travel	*T*_*C*_	Mobility	*E*_*C*_
Other	*T*_*C*_	Education	*E*_*C*_
		Service	*E*_*C*_
		Financing	*E*_*C*_
		Insurance	*E*_*C*_
		Other	*E*_*C*_

The classification of committed expenses (*E*_*C*_) follows Aschauer et al. (2019) as well as Mokhtarian and Chen (2004): expenses on goods associated with physical needs or maintenance were classified as ‘committed’. People need to eat (food), take care of their health (personal), and need a dwelling (housing) with equipment (furnishing). Further committed expenses are financing, insurance, services not related to leisure activities, education, and travel. Freely chosen expenses include out-of-home accommodation (mainly visiting a restaurant and holidays), leisure and recreational goods, as well as electronics and communication devices, which are mainly used for entertainment. ‘Clothing’ was also classified as ‘non-committed’ although it is at least partially essential. The reason is that clothing expenses add up to fairly high amounts in our sample, indicating that the ‘technical minimum’ is exceeded.

Those activity and expenditure categories which have been classified as ‘freely consumed’ as described above were further subdivided into two groups:

1. Categories that are entirely or at least predominantly freely consumed were classified as *T*_1_ and *E*_1_. *T*_1_ includes leisure; *E*_1_ includes leisure, accommodation (mainly eat outside), and electronic.
2. Categories that are committed by their nature, but it seems that most respondents have exceeded the technical minimum, were classified as *T*_2_ and *E*_2_. *T*_2_ includes eating and shopping; *E*_2_ includes clothes.

**Fig. 4:**
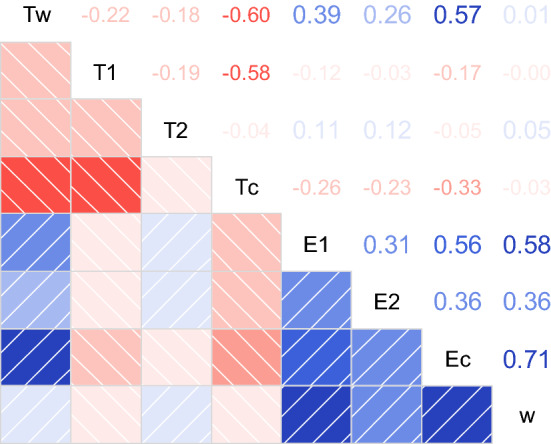
Correlogram of model variables

Figure 4 shows the correlation pattern of the model variables (descriptive statistics of these variables are provided in the “Appendix” section, Table 10). *T*_*W*_ is positively related with *E*_*C*_ and negatively with *T*_*C*_*—*as assumed in the theory and specified in Eq. (13). Another aspect to be noted is the opposite pattern of time-use and expenditure variables: all time-use variables are negatively correlated due to the common time constraint *τ*, whereas the expenditure variables are positively correlated among each other and also with *T*_*W*_. This follows from the equalizing effect of labour income: it increases with *T*_*W*_ and increases in turn the available budget for all kinds of goods.

The model estimation was carried out using a maximum likelihood estimation. It can be used under the normality assumption to estimate the parameters from the nonlinear equation system (13)–(15), which is re-written as:

18 $$ \hat{Y}_{i} = g_{i} \left( \beta \right) + \eta_{i} , i \in \left\{ {1, \ldots ,3} \right\} $$

where *g*_*i*_ denotes a function of parameter vector *β* and error terms $\eta_{i} \sim N\left( {\mu_{i} ,\sigma_{i} } \right)$. The joint density of all error terms can be expressed as:

19 $$ f\left( \eta \right) = f\left( {\eta_{1} } \right)f\left( {\eta_{2} |\eta_{1} } \right)f\left( {\eta_{3} |\eta_{1} ,\eta_{2} } \right) $$

The log-likelihood function of a sample of size *J* is:

20 $$ LL\left( \eta \right) = \sum\limits_{i = 1}^{J} {\log \left[ {f\left( {\eta_{1} } \right)f\left( {\eta_{2} |\eta_{1} } \right)f\left( {\eta_{3} |\eta_{1} ,\eta_{2} } \right)} \right]} $$

The maximum log-likelihood function in Eq. (20) yields estimates of the parameters in Eqs. (13)–(15). The VoL and VTAW can be calculated by entering these estimates in Eqs. (16) and (17). As stated in “Data preparation” section we tested two assumptions regarding how expenses are shared between members of the same household. The default dataset assumes ‘proportional expenses’ according to the labour income. The alternative dataset assumes ‘equal expenses on freely chosen goods’. This was achieved by allocating all expenses on freely consumed goods (*E*_1_ and *E*_2_) at equal amounts to the household members; the committed expenses (*E*_*C*_) were left unchanged (i.e. proportional to the labour income) to avoid negative disposable incomes (*E*_*C*_ > *wT*_*W*_), which can cause negative square roots in Eq. (13).

Table 4 shows the result of the estimation. The default dataset (proportional expenses) yields a VoL of 8.17 €/h, which is below the average wage of 12.14 €/h. As shown by many (see Jara-Díaz 2007 for a synthesis) the VoL equals the total value of work given by the wage plus the value of time assigned to work (VTAW). Therefore, the VTAW is negative with an average of − 3.97 €/h; it means that the average person works for the money and dislikes work as an activity. The alternative dataset (equal expenses on freely chosen goods) yields a VoL of 9.68 €/h, which is 18% higher. The difference arises from the lower estimate of *Φ* (0.308 vs. 0.365). The interpretation is straightforward: the implicit transfer of income between household members causes that the expenses have less statistical influence on the working time *T*_*W*_ [the response variable in Eq. (13)], because the expenses are equalised but *T*_*W*_ continues to differ between members of the same household. This results in a lower sensitivity of *T*_*W*_ with respect to changes in expenses and consequently in a lower value of income (*λ* and *Φ*). The sensitivity analysis gives an idea to what extent and in which direction the VoL is influenced by how resources are allocated among household members. Given the importance of this aspect, we consider household models (either in the cooperative or non-cooperative version) as an avenue for future work. The remaining results of this paper are based on the dataset with proportional expenses, because it yields a clear balance between labour income and expenses at the individual level and permits comparison with existing studies reported in Table 1, which have used samples of one-worker households (Jara-Díaz et al. 2016) or one-person-one-worker households (Konduri et al. 2011).

**Table 4:** Results of the model estimation from the total sample

Attribute	Proportional expenses		Equal expenses	
	Value	t-value	Value	t-value
*Model specification*				
# Persons	737		737	
# Parameters	4		4	
# Equations	3		3	
*Model parameters*				
$\theta_{w}$	− 0.421	− 4.94	− 0.217	− 4.90
$\theta_{1}$	0.732	172.70	0.735	172.92
$\theta_{2}$	0.268	–	0.265	–
$\varPhi$	0.365	13.88	0.308	19.02
$\varphi_{1}$	0.226	13.55	0.178	19.88
$\varphi_{2}$	0.139	–	0.130	–
*r-squares*				
*T*_*w*_ equation	0.702		0.712	
*T*_1_ equation	0.624		0.624	
*E*_1_ equation	0.379		0.096	
*Lagrange multipliers*				
*μ*	0.0277		0.0276	
*λ*	0.0043		0.0035	
Values of time (€/h)	Value	95% C.I.	Value	95% C.I.
*VoL *=* μ*/*λ*	8.17	[7.15, 9.19]	9.68	[8.91, 10.44]
Observed wage	12.14	[11.77, 12.50]	12.14	[11.77, 12.50]
*VTAW *=* VoL–w*	− 3.97	[− 4.99, − 2.94]	− 2.46	[− 3.23, − 1.69]

Please note that (1) the 95% confidence intervals of the VoL were estimated using the Delta method (Daly et al. 2012); (2) the parameters $\theta_{2}$ and $\varphi_{2}$ have no t-values, because they were not estimated but calculated as differences of other parameters: $\theta_{2} = \varTheta - \theta_{1}$ and $\varphi_{2} =\Phi  - \varphi_{1}$; (3) the parameter *Θ* is not estimated because it is set to one for normalisation, i.e., all other parameters are estimated in units of *Θ*

### Heterogeneity of the VoL across different population segments

A segmented consideration of the values of time seems to be important given the large differences between VoL estimates of different population segments in previous studies (see Table 1). These studies have consistently used a priori segmentation (i.e., the segments were treated as independent samples and separate models were estimated for each segment), but there are different options how to conduct a segmentation. These options have never been compared to each other, although they have different strengths and weaknesses and might yield different results.

In this section we compare alternative options to capture the heterogeneity in the VoL across seven segmentation variables, each of which was treated as follows: the variable was transformed to a binary variable (if not already binary) in a way that it identifies two groups of similar size (low vs. high age; low vs. high income etc.). Table 5 shows these variables along with their original distribution and binary segments.

**Table 5:** List of the variables used for segmentation

Variable	Original distribution	#	Binary segments	#
Urbanity	Urban	178	Urban	178
	Intermediate	227	Nonurban	559
	Rural	332		
Gender	Male	368	Male	368
	Female	369	Female	369
Age (years)	≤ 19	17	< 46	358
	20–29	50	≥ 46	379
	30–39	139		
	40–49	265		
	50–59	238		
	≥ 60	28		
Education	Compulsory school	23	< HS degree	289
	Apprenticeship	266	≥ HS degree	448
	High school degree	176		
	university	272		
# Children	0	467	None	467
	1	127	≥ 1	270
	2	124		
	≥ 3	19		
# Workers	1	157	One	157
	2	497	≥ 2	580
	3	58		
	≥ 4	25		
Pers. income [€/week]	< 200	53	< 432	374
	200–399	232	≥ 432	363
	400–599	269		
	600–799	121		
	≥ 800	62		

#### Segmentation approaches

The possible influence of the segmentation method on the VoL manifests in Eq. (16): it reveals the VoL as a function of four observed variables and one estimated parameter as follows:

- The VoL increases with observed wage rate *w*, working time *T*_*W*_, and committed time *T*_*C*_;
- It decreases with observed committed expenses *E*_*C*_ and the estimated parameter *Φ*.^9^

This means that the segmentation method can affect the VoL only through the parameter *Φ* because this is the only estimated quantity in Eq. (16). We compared three options of how to estimate *Φ*:

1. *Ex-Post Segmentation* The parameters are estimated from the total dataset using a global model. The VoL is then calculated from the segmented dataset based on the global *Φ* estimate; the VoL accounts only for differences in the distribution of observed variables, whereas *Φ* is constant across all segments.
2. *A-Priori Segmentation* The dataset is segmented beforehand; the parameters are estimated for each of the segmented datasets, which implies that all parameters are segment-specific. The VoL is then calculated from the segmented dataset based on segment-specific *Φ* estimates; it accounts for differences in the distribution of observed variables as well as other differences that affect the estimation of *Φ*.
3. *Interaction terms* The parameters are estimated from the total dataset using a model with interaction terms involving the segmentation variables. Each of the four main effect parameters (*θ*_*w*_, *θ*_1_, *φ*_1_, *Φ*) can have an interaction term independent from the other parameters. This way, segment-specific *Φ* values can be obtained and used to calculate the VoL for each segment. For the sake of comparability, we used the binary grouping variables also for the interaction model, although a moderator variable could, in principle, have a higher scale level (e.g., actual income rather than a binary dummy indicating low and high income).

#### Ex-post versus a-priori segmentation

**Fig. 5:**
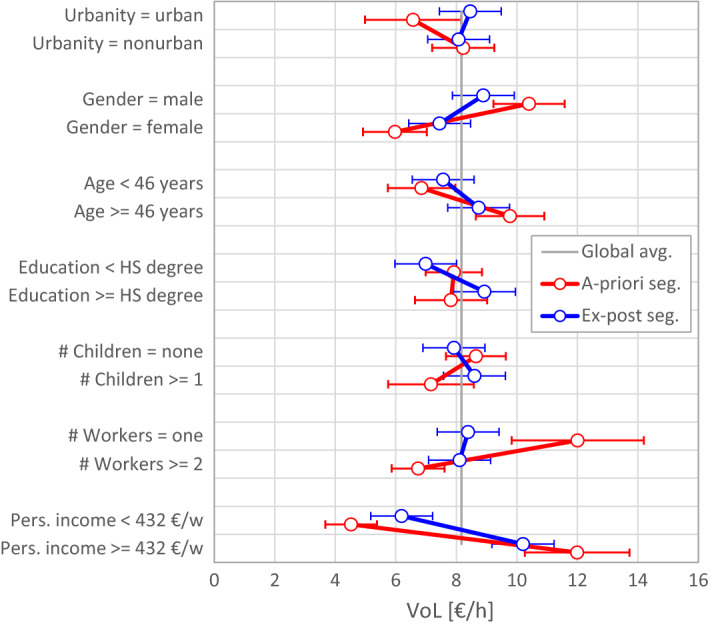
VoL estimates and 95% confidence intervals (according to the Delta method) of population segments, obtained from a priori segmentation and ex-post segmentation compared against the global average; oblique lines connect the VoL of two complementary segments

Figure 5 compares the segmented VoL estimates from ex-post segmentation (the most restrictive model where all parameters are estimated from the total sample) with those from a priori segmentation (the least restrictive model where all parameters are segment-specific); results are provided in the “Appendix” section, Table 11. Both methods yield similar results when segmenting by gender, age, and income—which suggests that these classifications have a consistent impact on the VoL. However, four segmentation variables yield very dissimilar or even reversed effects depending on the method used: urbanity, level of education, presence of children, and number of workers in the household. The reversed effect of the presence of children on the VoL provides no indication of the superiority of either procedure, because this effect can indeed be twofold as pointed out by Jara-Díaz et al. (2013): on the one hand, children require a lot of time, which translates into more time pressure compared to childless households; on the other hand, taking care of children can be a pleasurable activity for parents. However, the segmentation with respect to the educational level appears counter-intuitive in the a priori case, because the VoL of both low and high education segments deviates in the same direction from the global average.^10^

This raises the question why, in some cases, a priori segmentation causes these problems with reversed effects and counter-intuitive results. We found the main reason in the sensitivity of the VoL to the variance of the working time (*T*_*W*_) in each segment, which becomes effective only if the parameters are estimated segment-specific (i.e. in the case of a priori segmentation). This sensitivity can be intuitively explored from the behaviour of the *T*_*W*_ in Eq. (13) during the process of parameter estimation:

- A large variability of *T*_*W*_ must be reflected by a large variability of the predicted working time *T*_W_* to achieve a close fit, which means large responses of *T*_W_* to given changes in the explanatory variables^11^;
- The responsiveness of *T*_W_* is larger, if *θ*_*w*_ (baseline utility of work) is more negative. The reason is the right term under the square root in Eq. (13) because this term increases linearly with − *θ*_*w*_^12^;
- A negative *θ*_*w*_ enforces a large *Φ* (baseline utility of freely consumed goods) to satisfy the condition that the marginal utility of work plus labour income equals the marginal utility of leisure as defined in Eq. (6).

**Fig. 6:**
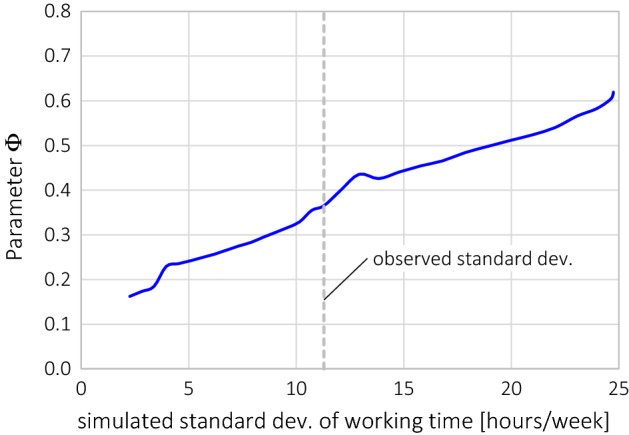
VoL and parameter *Φ* of a model series estimated from simulated samples, in which the variance of *T*_*W*_ and *T*_*C*_ was changed systematically; all mean values remained unchanged

In order to verify this in our data, Fig. 6 shows the results of a simulation. We generated a series of datasets based on the total sample (n = 737). In each dataset we pivoted the values of *T*_*W*_ symmetrically around the mean, such that the mean does not change, but the variance becomes smaller or larger; the changes in *T*_*W*_ were balanced by opposite changes of *T*_*C*_ to meet the time constraint; everything else was left unchanged. The result shows the close response of the parameter *Φ* to changes in the variance of *T*_*W*_ in line with the aforementioned description; it

**Fig. 7:**
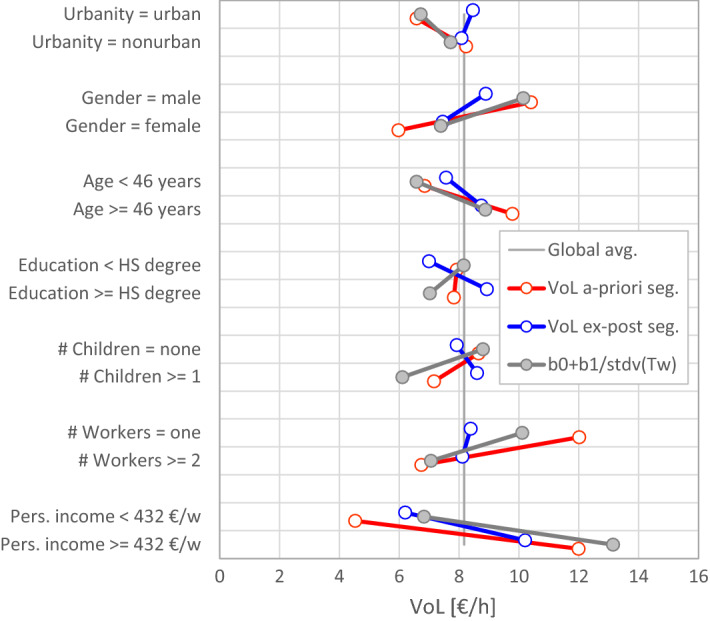
Influence of the inverse variance of *T*_*W*_ on the VoL estimates obtained from a priori segmentation

Figure 7 shows how this mechanism affects the segmentation results. The blue and red lines are the same as in Fig. 5; the grey line shows the inverse standard deviation of *T*_*W*_ in each segment.^13^ The deviation of the a priori segments from the ex-post segments throughout follows the direction of the grey line, especially in the three cases of reversed results (urbanity, education, and presence of children). The sensitivity to the variance of *T*_*W*_ makes a priori segmentation vulnerable to unexpected external influences because the variance of *T*_*W*_ can differ for many reasons. An example is the segmentation by gender. Men are more often full-time employed (high *T*_*W*_ but small variance of *T*_*W*_), whereas women have more flexible part-time arrangements (low *T*_*W*_ but large variance of *T*_*W*_). The lower *T*_*W*_ (and lower wage) of women causes a lower VoL; this is already captured in the ex-post segment. The a priori segment yields an even lower VoL for women, because it accounts for the larger variance of womens’ *T*_*W*_. Does this really indicate a low value of time? Or rather the opposite: a higher time pressure on women resulting from unpaid duties such as domestic work and child care, which requires more flexibility with paid work? The same pattern applies to the particularly high VoL of single workers in the a priori segment: single workers are in most cases full time workers with large *T*_*W*_ and small variance of *T*_*W*_ The ex-post segmentation yields almost no difference, because the larger *T*_*W*_ is balanced by a slightly lower wage of single workers, and the influence of the variance of *T*_*W*_ disappears.

To summarize, we find that a priori segmentation yields, for some segments, peculiar results and reversed effects compared to those of ex-post segmentation. We have presented an explanation for these problems based on the role played by the variance of working time within each segment. An additional problem of a priori segmentation can be large standard errors, if the underlying segments have a small sample size.

#### Interaction terms versus ex-post segmentation

The problems associated with a priori segmentation call for a parsimonious use of degrees of freedom—such as reflected by the use of interaction terms, which allow for more flexibility from single interaction terms up to a full interaction model.^14^ In preparation of this approach we modified the model Eqs. (13–15) by replacing each instance of the main effect parameter with an interaction term of the form $\beta_{i} Z^{{y_{i} }}$, where $\beta_{i}$ denotes the main effect parameter, Z the segmentation variable, and $y_{i}$ the interaction parameter, which gives the sensitivity of $\beta_{i}$ with respect to changes in *Z*. We estimated all 15 possible interaction models for each segmentation variable, but only 5 models are shown in the “Appendix” section (Table 11): four ‘single interaction models’ with one interaction term on one of the four main effect parameters and a ‘full interaction model’ with interaction terms on all four parameters.

**Fig. 8:**
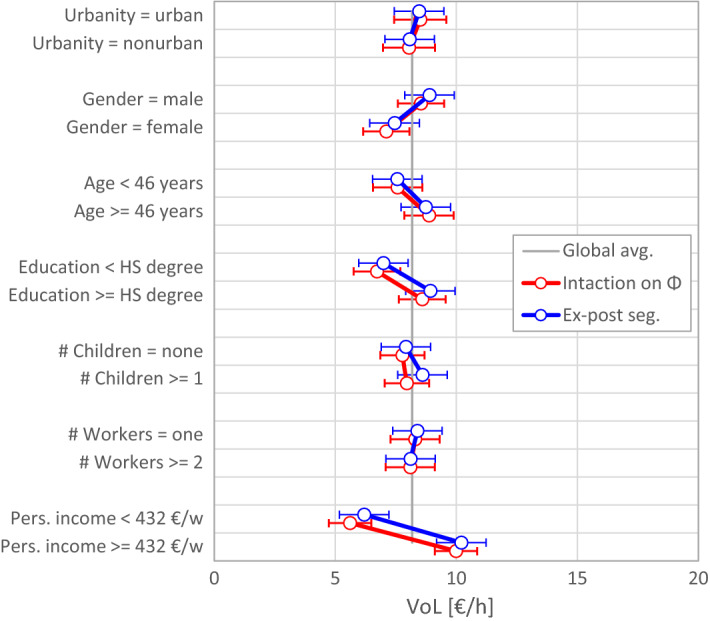
VoL estimates of different population segments gained from ex-post segmentation and from as single interaction model with an interaction term on *Φ*

The full interaction model has the same degrees of freedom as a priori segmentation; it yields indeed very similar results. The single interaction models are more similar to ex-post segmentation. Those models with an interaction term on another parameter than *Φ* can only change the magnitude of the VoL in both segments but not the ratio between the two segments, unless *Φ* has also an interaction term. An interaction term on *Φ* causes the largest deviation from ex-post segmentation. Figure 8 compares the *Φ*-interaction model with ex-post segmentation. It reveals only one noticeable (but still insignificant) difference for households with and without children.

Since the VoL is a latent variable which cannot be observed, there is no basis for comparison across models based on the VoL estimates themselves. However, from the analysis above, we conclude that a priori segmentation and the full interaction model are not appropriate in our case. The large number of degrees of freedom makes the estimation process sensitive to the variance of working time within each segment, to which ex-post segmentation is not sensitive. This difference is evident in the simulation results (Fig. 6), empirical results (Fig. 7), and in the behaviour of Eq. (13). We recommend a limited number of degrees of freedom to make the estimation process more robust against the influence of the working-time variance. The most restrictive option is ex-post segmentation which supresses this influence entirely. From our results, however, it seems that a single interaction term on the parameter *Φ* can be used to account for heterogeneity in the sample without seriously affecting the robustness of the model.

### Value of travel time saving (VTTS) and value of time assigned to travel (VTAT)

As explained earlier, the value of travel time savings (VTTS)—estimated from travel choice models—represents the willingness-to-pay to diminish travel time by one unit. As originally shown by DeSerpa (1971), the VTTS has two components: the opportunity cost regarding other activities (leisure or work) and the value of a reduction of the travel activity by itself. The first component is the value of leisure (VoL). The second-called the value of time assigned to travel (VTAT)—depends on the travel conditions. Analytically, the formula is

21 $$ VTTS_{m} = VoL - VTAT_{m} $$

where VTTS_m_ is the (mode-specific) value of travel time saving estimated from a travel choice model, VoL is the (individual-specific) value of leisure, and VTAT_m_ is the value of time assigned to travel, driven by mode-specific characteristics such as comfort and how productively in-vehicle time can be used for secondary activities (for a general derivation see Jara-Díaz 2007, Chapter 2). Equation (21) shows that unless one has an estimate of the opportunity cost of travel given by the VoL, the VTAT_m_ simply cannot be estimated. As explained earlier, this is exactly the reason why a time-use model is needed. The VoL estimates were presented in “Results” section of this paper, while the mode-specific VTTS_m_ were estimated in a parallel effort by Schmid et al. (2018) from a model which combines different data types (RP, SP) and experiment types (mode, route, and shopping destination choice) using 21,681 choice observations of 744 respondents. The data used for the travel-choice model originates from the same MAED survey, which was used for the continuous choice models in this paper.^15^ The SP data were collected by a follow-up survey from a subsample of 504 respondents. A mixed logit model was estimated, which accounts for unobserved heterogeneity in the VTTS and the availability of the different modes and includes scale parameters for the different data and experiment types (see e.g. Train 2009).

The common data source makes the VoL and VTTS_m_, although estimated separately, compatible to each other. However, a consequence of the independent estimation is that possible correlations between the error terms of continuous decisions and discrete mode choices are not considered. Munizaga et al. (2008) tested the effect of a joint estimation of both types of decisions using full information maximum likelihood (FIML) in comparison to an independent estimation of both types. They had very large correlations between continuous and discrete choices (up to 0.676), possibly because they used a sample of long-distance commuters, for whom the chosen travel mode can make a substantial difference on how their day is organised. Despite the large correlations, they found only small differences between the parameters from joint and independent estimations. Table 6 shows the correlations between the error terms of the continuous equations and the mode choice probabilities estimated from the MAED sample.^16^ They are much smaller than those reported by Munizaga et al. (2008); the largest is 0.108 between the error term of working time and the choice probability of public transport. It indicates that the bias from ignoring the correlations between continuous and discrete decisions is likely to be small. Future work might include a joint estimation of continuous and discrete decisions.

**Table 6:** Pairwise correlations between error terms of continuous equations and Lee-transformed choice probabilities of the mode choice model

Error term	*Φ*^−1^ (P_walk)	*Φ*^−1^ (P_bike)	*Φ*^−1^ (P_car)	*Φ*^−1^ (P_public)
Error (*T*_*W*_)	− 0.036	0.022	− 0.016	0.108
Error (*Tf*_1_)	− 0.002	− 0.047	0.032	− 0.056
Error (*Ef*_1_)	0.049	0.077	− 0.066	0.106

**Fig. 9:**
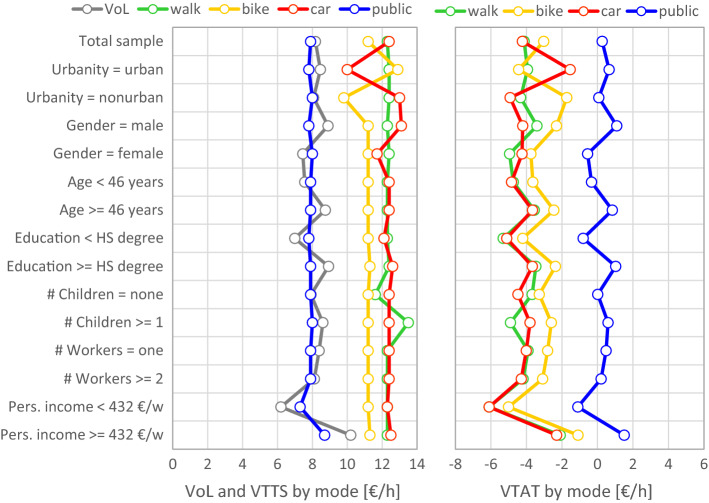
Value of leisure (VoL), mode-specific values of travel time savings (VTTS) and values of time assigned to travel (VTAT) for the total sample (top row) and for different population segments. Note that the VTTS estimates of the segments by age and number of workers in the household are equal to the global VTTS, because the mode choice model revealed insignificant interaction effects for the corresponding segmentation variables

**Fig. 10:**
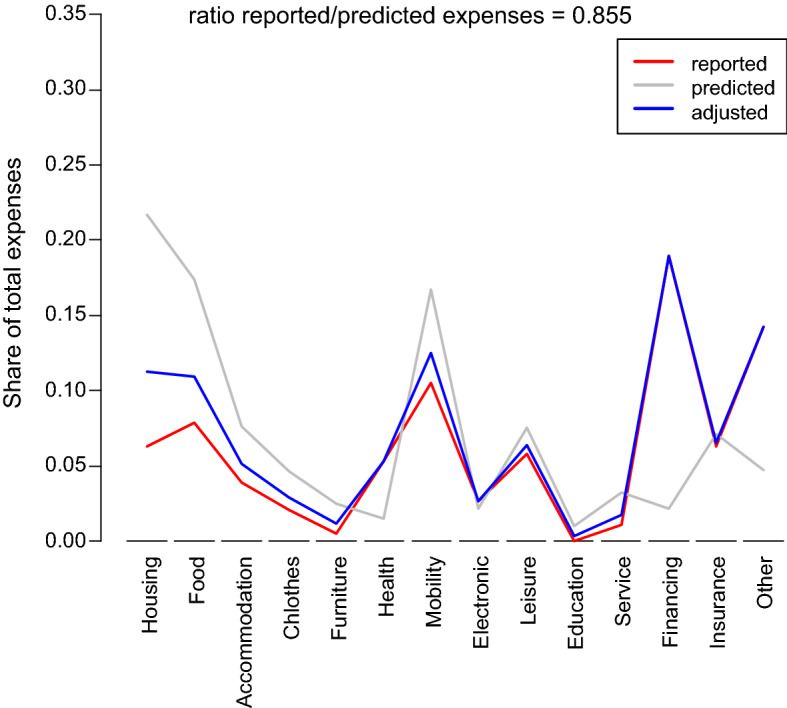
Example for the adjustment of reported expenditures to predicted expenditures (calculated as reported income minus predicted savings obtained from the model in Table 8) using the predicted expenditure shares (gained from the model in Table 9) as the benchmark

Figure 9 shows the VoL, VTTS, and VTAT estimates for different population segments; the VTTS and VTAT also for different travel modes (results are provided in the “Appendix” section, Table 12). The VTTS related to public transport is throughout lower than that of other modes including the car, which confirms a common finding (see Table 1 in Schmid et al. 2018). From Eq. (21) one can see that, for a given individual (i.e. a given value of leisure), the low willingness to pay to reduce travel time in public transport is caused by a large (predominantly non-negative) value of time assigned to public transport, as shown on the right hand side of Fig. 9. Another important aspect is the large difference between the VTAT of car and public transport (4.4 €/h on average), which persists even after controlling for user characteristics. The smallest difference arises in the urban segment with 2.2 €/h.

The findings regarding VTAT are indeed novel and interesting. They emerge exactly due to the possibility of disentangling the two components behind the VTTS. The main finding is that travel conditions in public transport (captured only by VTAT) are perceived more pleasant than those in a car, which seems to capture well the quality of service of public transport in Austria, contradicting the common opinion that traveling by car is generally more pleasant. We have no basis for comparison, because these are the first mode-specific VTAT estimates. But there are reasonable arguments why public transport users might perceive the time assigned to travel more pleasant than car drivers (and are therefore less time-sensitive): they are released from the driving task and can engage in many kinds of secondary activities, which makes the time assigned to travel more comfortable, entertaining, and useful. Flügel (2014) provides a summary of why public transport travellers may be less time-sensitive than car travellers.

## Synthesis and conclusions

The aim of this study was to obtain representative estimates for the value of leisure (VoL), value of time assigned to work (VTAW), and (for the first time) mode-specific values of time assigned to travel (VTAT) of Austrian workers. VTAT have been obtained by comparing the VoL with mode-specific values of travel time savings (VTTS) from a related study based on the same data source (Schmid et al. 2018).

The average VoL in the population was estimated at 8.17 €/h. This is considerably less than the average wage rate of 12.14 €/h; the result is a negative VTAW of − 3.97 €/h, indicating that time assigned to work is valued negatively on average and people work mainly for the salary. The result seems reasonable in the sense that the VoL is not too far away from (but also not identical to) the wage rate. In their estimations of the VTTS, Schmid et al. (2018) found that the mode-effect dominates over the effect of user characteristics; the average VTTS estimates for walk, bike, car, and public transport are 12.30, 11.20, 12.40, and 7.90 €/h, respectively. An important implication is that the direct utility of time assigned to travel, expressed by the VTAT, has inverse signs for different modes: it is strictly negative for walking, cycling and car driving, and close to zero (predominantly positive) for public transport with an average of 0.27 €/h. The clear priority of public transport has not been identified in previous studies (e.g. Wardman 2004; Shires and De Jong 2009). It may indicate that the public transport benefits more than other modes from technological innovations and mobile devices such as smartphones, etc. These devices affect the perceived comfort and how in-vehicle time can be used for secondary activities such as work, communication, or entertainment. From a transport planning perspective, the results support those who claim that the conditions of travel matter greatly (e.g. Litman 2008; Lyons et al. 2013; Flügel 2014) and investments in better travel conditions are as important as investments in higher speed to attract customers to public transport.

An important finding with respect to the reliability of the VoL is its sensitivity to the variance of working time in the sample: a high variance causes a low VoL and vice versa. This has not been noted in previous studies, possibly because it is not visible in the equations but results from a specific behaviour of the equations during parameter estimation. This has several implications:

- It might be responsible for some of the fluctuations of VoL estimates in previous results (see Table 1).
- It can cause biased VoL estimates of population segments if the variance of working time in the segment deviates from the global average. This problem appears only if a priori segmentation is used (i.e., if separate models are estimated for each segment). To be on the safe side, we recommend using ex-post segmentation (i.e., estimation of global parameters and calculation of the VoL in the segmented data with these parameters). In our sample it seems that single interaction terms in the global model do not seriously affect the robustness.
- It might affect the comparison of countries with different degrees of regulation of the labour market. Part-time workers exhibit a large variability in any labour market, but the variability of full-time workers depends on the degree of regulation. Full-time workers in a strongly regulated market (as in Austria and many other European countries) exhibit a low variance in working time because the maximum is limited by collective agreements, whereas full-time workers in a de-regulated market may exhibit a larger variability.

To the best of our knowledge, this is the first study that uses a data source which has been collected with the explicit intention to estimate all components of the time-use framework introduced by Jara-Díaz and Guevara (2003): a representative sample, where all information required for modelling has been collected from the same individuals at the same time in a diary-based format. Given the high data quality and the fact that we obtained reasonable results (in terms of a plausible size and moderate variability of VoL estimates) we conclude:

- Going ahead towards practical usability of the values of time obtained from the time-use framework model not only requires advanced models, but (possibly even more so) advanced data.
- If high-quality data is used for parameter estimation, the data collection effort seems to be rewarded by more reliable results. This interpretation should be confirmed by further efforts into gathering of high-quality data.

A data-collection technique that is likely to become more important in the future is probabilistic merging. It would thus be a promising option for further research to compare the MAED data with an artificial dataset in which the expenses are imputed from the latest Austrian consumer expenditure survey. This might answer the question how much is lost by probabilistic merging compared to simultaneous collection—which is indeed more burdensome.

## Appendix

**Table 7:** Linear models for adjustment of activity assignments; the parameters indicate how 1 h less of work is replaced by additional time spent on other activities (and vice versa) to meet the time constraint; *left side*: adjustment model for the increase in working time (if reported working time was lower than effective working time); *right side*: adjustment model for reduction of working time (if reported working time was higher than effective working time)

Activity	Increase of working time			Reduction of working time		
	Parameter	t-value	*p* value	Parameter	t-value	*p* value
Travel	0.166	3.586	0.000	0.000	–	–
Sleep	0.083	1.393	0.164	0.019	0.172	0.863
Eating	0.143	3.274	0.001	0.053	0.731	0.465
Education	0.076	1.664	0.097	0.075	1.190	0.235
Personal	0.239	3.383	0.001	0.000	–	–
Domestic	0.000	–	–	0.430	2.056	0.041
Shopping	0.008	0.461	0.645	0.017	0.497	0.619
Leisure	0.285	2.483	0.013	0.406	1.944	0.053

The two models in Table 7 correspond to our assumption that an incidental increase of working time beyond the usual level (left model) causes different re-arrangement patterns than an incidental reduction below the usual level (right model). Furthermore, we assume strictly substitutional relationships between work and other activities, such that an increase of working time causes a reduction of other activities and vice versa. This was informed by restricting the lower bound of the parameters to zero. In three cases, we obtained parameters at the lower bound, because the estimated values were negative, although they did not significantly differ from zero: (1) Domestic work is not reduced as the working time increases. This might indicate that domestic activities cannot be reduced easily due to their strongly committed nature. (2) Travel is not increased as the working time decreases. This makes sense due to the complementary relationship between work and the travel to work. The parameter might indeed be negative, but it is fixed here to zero because of the insignificant deviation. (3) Personal activities are also not increased as the working time decreases. This might indicate that an additional demand for personal activities is usually not the reason why the working time is reduced below its usual level.

Figure 10 illustrates by means of an example (a particular person) how the reported expenses were adjusted to match the balance between income and expenses, while keeping the inter-person variability of expenses. The aim of the adjustment is to equal the total expenses (sum of reported expenses) to the predicted expenses such that the ratio reported/predicted is one, irrespective of whether or not the reported expenditure shares match the predicted shares. The person in Fig. 10 reported only 85.5% of predicted expenses (obtained as income minus predicted savings from the savings model in Table 8). The missing 14.5% were imputed by increasing those expenditure shares that were below the predicted shares obtained from the expenditure shares model in Table 9 (in this case: Housing, Food, Accommodation, Clothes etc.). The remaining shares, which equal or exceed the predicted shares, were not increased (Health, Electronic, Financing, Other). Please note that the predicted shares (grey line) are already adapted to the individual’s household and personal characteristics. The adjustment works inversely for persons whose reported income exceeds the predicted income (ratio reported/predicted > 1).

**Table 8:** Linear model for prediction of savings using household and personal characteristics as predictors (R^2^ = 0.269, F-statistic = 9.209, *p* value = 0.000)

Predictor	Estimate	t-value	*p* value
Intercept	23.741	0.403	0.687
Workers in household	48.825	5.855	0.000
Children in household 6–14 years	− 7.218	− 0.920	0.358
Region = Styria	− 18.977	− 1.501	0.134
Region = Carinthia	− 27.208	− 1.308	0.191
Persons in household	− 19.224	− 3.139	0.002
Region = East Austria	19.283	1.777	0.076
Home = one or two family	− 10.746	− 1.125	0.261
Total income per person week	0.292	10.883	0.000
Gender = female	15.039	1.426	0.154
Age	− 1.182	− 2.569	0.010
Education = HS degree	− 19.872	− 1.895	0.059
Workplace urbanity = urban	− 47.698	− 2.669	0.008
Workplace urbanity = rural	− 36.196	− 1.682	0.093
Workplace urbanity = intermediate	− 30.971	− 1.560	0.119
Work time actually	− 1.631	− 3.094	0.002
Employment = self employed	− 23.485	− 1.440	0.150
PT pass = other discount	− 36.923	− 2.991	0.003
PT pass = railway discount	18.554	0.910	0.363
Number of trips of person per week	− 2.518	− 3.224	0.001
Trip duration of person per week	− 0.055	− 3.009	0.003
# Activities of person per week	0.427	1.238	0.216
Share of mobile days of person	40.607	0.828	0.408
Survey wave	16.905	1.854	0.064
Activity duration: shopping	− 9.943	− 3.782	0.000
Activity duration: other	5.121	1.723	0.085
Activity duration: personal	− 0.567	− 0.876	0.382
Activity duration: domestic	0.660	1.194	0.233

**Table 9:** Estimated parameters of a multinomial logit model for prediction of expenditure shares by category using household and personal characteristics as predictors; reference category = ’other expenditures’ (McFadden’s Pseudo-R^2^ = 0.130 compared to the constants only model)

Predictor	Housing	Food	Accommod.	Clothes	Furniture	Health	Mobility	Electronics	Leisure	Education	Service	Finances	Insurance
Intercept	4.469	2.896	− 0.100	1.696	1.870	− 0.512	1.811	1.232	1.751	0.053	− 1.045	1.067	1.479
# Adults in household	− 0.191	0.034	− 0.195	− 0.082	− 0.203	− 0.023	− 0.033	− 0.112	− 0.054	− 0.125	− 0.187	− 0.168	− 0.120
# Children in household < 6 years	0.046	0.101	− 0.158	− 0.090	0.065	0.018	− 0.055	− 0.214	− 0.189	− 0.195	− 0.084	0.132	0.042
# Workers in household	− 0.014	0.083	0.086	0.034	0.273	− 0.132	0.014	0.265	− 0.064	− 0.107	0.130	− 0.394	0.056
Region = upper Austria	− 0.213	0.063	0.150	0.174	− 0.256	− 0.103	− 0.259	− 0.224	0.000	− 0.177	0.077	− 0.259	− 0.090
Region = Styria	0.072	0.074	− 0.003	0.121	0.037	0.221	− 0.060	0.134	0.009	− 0.039	− 0.050	0.018	0.238
Region = Salzburg	0.719	0.745	0.762	0.541	0.530	0.544	0.667	0.815	0.592	0.922	1.086	0.990	0.894
Region = Carinthia	− 0.301	− 0.119	0.302	− 0.298	− 1.176	− 0.299	− 0.311	− 0.315	− 0.421	0.302	− 0.261	0.397	− 0.211
Age	− 0.013	− 0.009	− 0.018	− 0.020	− 0.007	0.004	− 0.007	− 0.006	− 0.011	− 0.005	0.000	− 0.004	− 0.007
Education = apprenticeship, college	0.054	0.085	0.013	0.059	0.144	− 0.121	0.041	0.088	− 0.036	0.173	0.005	− 0.129	0.143
Gender = female	− 0.018	− 0.098	− 0.025	− 0.157	− 0.041	0.019	− 0.048	− 0.197	0.018	0.010	0.163	0.038	− 0.044
Residence urbanity = rural	− 0.125	− 0.062	− 0.281	0.067	0.074	0.170	0.067	− 0.077	− 0.142	− 0.024	− 0.092	− 0.230	0.126
Household job income/week	0.000	0.000	0.000	0.001	0.000	0.000	0.000	0.000	0.001	0.000	0.000	0.001	0.000
Regular working time	− 0.020	− 0.012	− 0.014	− 0.010	− 0.004	− 0.016	− 0.014	− 0.015	− 0.010	− 0.022	− 0.009	− 0.026	− 0.010
License available	0.115	− 0.012	0.063	− 0.013	0.097	− 0.028	0.032	− 0.126	0.019	− 0.051	0.189	0.022	0.065
PT pass = other discount	− 0.009	− 0.132	0.034	− 0.246	− 0.179	0.150	− 0.093	0.007	− 0.134	0.263	0.094	− 0.272	− 0.016
PT pass = railway discount	0.386	0.590	0.563	0.404	0.146	0.421	0.463	0.357	0.370	0.364	0.131	0.332	0.320
# Activities in household/week	0.008	0.008	− 0.003	0.011	0.011	0.009	0.007	0.011	0.003	0.018	0.015	0.003	0.002
Share of mobile days in household	0.292	0.245	2.678	0.220	− 1.022	1.318	0.579	0.685	0.539	1.381	1.398	0.694	0.375
Modal share of car in household	0.108	0.302	− 0.045	− 0.056	0.183	0.332	0.528	− 0.182	0.188	0.093	0.440	0.427	0.427
Trip distance in household/week	0.000	0.000	0.000	0.000	0.000	0.001	0.000	0.000	0.000	− 0.001	0.000	0.000	0.000
Trip duration in household/week	0.000	0.000	0.000	0.000	0.000	0.000	0.000	0.000	0.000	0.000	0.000	0.001	0.000
# Trips in household/week	− 0.047	− 0.033	− 0.024	− 0.038	− 0.035	− 0.021	− 0.037	− 0.039	− 0.027	− 0.034	− 0.038	− 0.048	− 0.029
Commuting distance	− 0.003	− 0.003	− 0.002	− 0.009	− 0.001	0.001	0.000	− 0.008	− 0.007	− 0.005	− 0.004	− 0.005	− 0.006
Survey wave	− 0.320	− 0.451	− 0.216	− 0.331	− 0.602	− 0.401	− 0.233	− 0.119	− 0.277	− 0.251	− 0.270	− 0.504	− 0.208
Activity duration: eating	− 0.021	− 0.034	0.043	− 0.009	− 0.014	− 0.005	− 0.018	− 0.019	− 0.013	− 0.010	− 0.032	0.002	− 0.016
Activity duration: leisure	− 0.008	− 0.004	− 0.007	0.000	− 0.011	− 0.015	− 0.003	− 0.007	0.000	− 0.028	− 0.006	0.002	− 0.001
Activity duration: shopping	− 0.113	− 0.043	− 0.115	0.037	− 0.009	− 0.099	− 0.100	− 0.102	− 0.123	− 0.157	− 0.101	− 0.190	− 0.109

**Table 10:** Model variables along with their mean values and standard deviation (in brackets) across different population segments (time use variables: h/week; expenditures: €/week, wage: €/h)

Segment	n	*T*_*W*_	*T*_1_	*T*_2_	*T*_*C*_	*E*_1_	*E*_2_	*E*_*C*_	*w*
Total sample	737	37.8 (11.3)	28.9 (11.1)	11.4 (4.6)	89.8 (13.4)	80.0 (50.0)	26.3 (22.1)	332.4 (161.7)	12.14 (5.09)
Urbanity = urban	178	39.2 (12.0)	27.0 (11.6)	11.8 (4.3)	90.1 (12.6)	95.4 (54.9)	28.4 (23.9)	343.2 (148.0)	12.53 (5.00)
Urbanity = nonurban	559	37.4 (11.0)	29.6 (10.8)	11.3 (4.7)	89.7 (13.7)	75.1 (47.3)	25.6 (21.5)	329.0 (165.8)	12.01 (5.12)
Gender = male	368	42.3 (9.3)	29.8 (11.6)	11.3 (4.8)	84.6 (11.6)	93.5 (53.9)	30.8 (25.1)	394.7 (175.4)	12.76 (5.79)
Gender = female	369	33.3 (11.3)	28.1 (10.5)	11.6 (4.3)	95.0 (13.1)	66.5 (41.5)	21.8 (17.7)	270.3 (117.9)	11.51 (4.20)
Age < 46 years	358	35.9 (12.1)	29.0 (11.6)	11.5 (4.6)	91.6 (14.2)	70.7 (44.9)	25.5 (21.7)	298.2 (144.3)	11.42 (4.63)
Age ≥ 46 years	379	39.7 (10.1)	28.9 (10.6)	11.4 (4.5)	88.0 (12.4)	88.7 (52.9)	27.0 (22.6)	364.8 (170.6)	12.82 (5.41)
Education < HS degree	289	37.6 (10.7)	30.4 (10.5)	11.1 (4.4)	88.9 (13.4)	62.8 (33.9)	23.3 (21.0)	285.9 (120.6)	10.35 (3.38)
Education ≥ HS degree	448	38.0 (11.7)	28.0 (11.3)	11.7 (4.7)	90.3 (13.4)	91.1 (55.3)	28.3 (22.6)	362.5 (177.2)	13.29 (5.65)
# Children = none	467	39.3 (10.2)	29.6 (11.1)	11.2 (4.6)	87.9 (12.3)	81.8 (49.0)	26.0 (21.2)	324.7 (157.6)	11.80 (5.04)
# Children ≥ 1	270	35.4 (12.6)	27.7 (11.1)	11.9 (4.6)	93.0 (14.6)	76.9 (51.4)	26.8 (23.7)	345.9 (168.1)	12.72 (5.15)
# Workers = one	157	40.9 (9.3)	28.8 (11.3)	11.2 (4.3)	87.1 (12.3)	89.9 (50.3)	28.9 (24.1)	364.2 (150.2)	12.05 (4.95)
# Workers ≥ 2	580	37.0 (11.6)	29.0 (11.1)	11.5 (4.6)	90.5 (13.6)	77.3 (49.6)	25.6 (21.5)	323.9 (163.8)	12.16 (5.14)
Pers. income < 432 €/w	374	32.8 (11.9)	29.7 (10.9)	11.7 (4.5)	93.8 (14.2)	52.6 (26.2)	18.6 (15.5)	233.9 (95.9)	9.46 (2.83)
Pers. income ≥ 432 €/w	363	43.1 (7.8)	28.1 (11.2)	11.2 (4.6)	85.6 (11.1)	108.2 (52.9)	34.2 (25.0)	434.0 (152.7)	14.89 (5.43)

**Table 11:** Values of leisure estimated from different models (ex-post segmentation without and with interaction terms, a priori segmentation) across different population segments (€/h; in brackets: standard errors)

Segment	Ex-post segmentation with interaction terms on the following parameters:						A priori segmentation
	None	*Φ*	*θ*_*W*_	*θ*_1_	*ϕ*_1_	All four	
Total sample	8.17 (0.52)	8.16 (0.55)	8.02 (0.42)	7.89 (0.49)	7.98 (0.49)	8.10 (0.45)	
Urbanity = urban	8.46 (0.52)	8.51 (0.55)	9.09 (0.42)	8.18 (0.49)	8.26 (0.49)	6.19 (0.45)	6.57 (0.81)
Urbanity = nonurban	8.08 (0.52)	8.04 (0.55)	7.68 (0.42)	7.80 (0.49)	7.89 (0.49)	8.70 (0.45)	8.23 (0.52)
Gender = male	8.89 (0.52)	8.54 (0.49)	9.73 (0.44)	9.03 (0.55)	9.27 (0.58)	9.97 (0.44)	10.40 (0.60)
Gender = female	7.45 (0.52)	7.11 (0.49)	7.49 (0.44)	7.59 (0.55)	7.83 (0.58)	7.12 (0.44)	5.97 (0.54)
Age < 46 years	7.56 (0.52)	7.58 (0.52)	7.48 (0.49)	7.55 (0.52)	7.41 (0.51)	7.47 (0.58)	6.85 (0.57)
Age ≥ 46 years	8.74 (0.52)	8.87 (0.52)	9.36 (0.49)	8.73 (0.52)	8.57 (0.51)	8.91 (0.58)	9.78 (0.58)
Education < HS degree	6.99 (0.52)	6.72 (0.49)	6.82 (0.51)	7.00 (0.52)	6.61 (0.48)	8.80 (0.38)	7.92 (0.47)
Education ≥ HS degree	8.93 (0.52)	8.60 (0.49)	8.90 (0.51)	8.93 (0.52)	8.44 (0.48)	7.31 (0.38)	7.82 (0.61)
# Children = none	7.92 (0.52)	7.77 (0.47)	8.62 (0.43)	7.65 (0.49)	7.58 (0.49)	8.75 (0.41)	8.65 (0.51)
# Children ≥ 1	8.60 (0.52)	7.96 (0.47)	7.77 (0.43)	8.32 (0.49)	8.24 (0.49)	6.95 (0.41)	7.16 (0.72)
# Workers = one	8.39 (0.52)	8.30 (0.52)	9.28 (0.41)	8.41 (0.52)	8.56 (0.56)	11.37 (0.39)	12.01 (1.12)
# Workers ≥ 2	8.11 (0.52)	8.10 (0.52)	7.98 (0.41)	8.13 (0.52)	8.29 (0.56)	6.96 (0.39)	6.74 (0.44)
Pers. income < 432 €/w	6.19 (0.52)	5.61 (0.45)	5.53 (0.60)	5.93 (0.49)	5.92 (0.49)	7.60 (0.42)	4.52 (0.44)
Pers. income ≥ 432 €/w	10.20 (0.52)	9.99 (0.45)	11.84 (0.60)	9.80 (0.49)	9.78 (0.49)	9.76 (0.42)	11.99 (0.88)

**Table 12:** Values of leisure (VoL), values of travel time savings (VTTS) and values of time assigned to travel (VTAT) across different population segments; VTTS and VTAT also across travel modes (all in €/h)

Segment	VoL	VTTS				VTAT			
		Walk	Bike	Car	Public	Walk	Bike	Car	Public
Total sample	8.17	12.30	11.20	12.40	7.90	− 4.13	− 3.03	− 4.23	0.27
Urbanity = urban	8.46	12.40	12.90	10.00	7.80	− 3.94	− 4.44	− 1.54	0.66
Urbanity = nonurban	8.08	12.40	9.80	13.00	8.00	− 4.32	− 1.72	− 4.92	0.08
Gender = male	8.89	12.30	11.20	13.10	7.80	− 3.41	− 2.31	− 4.21	1.09
Gender = female	7.45	12.40	11.20	11.70	8.00	− 4.95	− 3.75	− 4.25	− 0.55
Age < 46 years	7.56	12.30	11.20	12.40	7.90	− 4.74	− 3.64	− 4.84	− 0.34
Age > = 46 years	8.74	12.30	11.20	12.40	7.90	− 3.56	− 2.46	− 3.66	0.84
Education < HS degree	6.99	12.30	11.20	12.10	7.80	− 5.31	− 4.21	− 5.11	− 0.81
Education > = HS degree	8.93	12.40	11.30	12.60	7.90	− 3.47	− 2.37	− 3.67	1.03
# Children = none	7.92	11.60	11.20	12.40	7.90	− 3.68	− 3.28	− 4.48	0.02
# Children > = 1	8.60	13.50	11.20	12.40	8.00	− 4.90	− 2.60	− 3.80	0.60
# Workers = one	8.39	12.30	11.20	12.40	7.90	− 3.91	− 2.81	− 4.01	0.49
# Workers > = 2	8.11	12.30	11.20	12.40	7.90	− 4.19	− 3.09	− 4.29	0.21
Pers. income < 432 €/w	6.19	12.30	11.20	12.30	7.30	− 6.11	− 5.01	− 6.11	− 1.11
Pers. income > = 432 €/w	10.20	12.30	11.30	12.50	8.70	− 2.10	− 1.10	− 2.30	1.50

## Footnotes

1 For the VTTS to represent the opportunity cost of travel, it must include all utility components, which are experienced while travelling, e.g. more comfortable seats or the availability of WIFI—but no one-off effects such as the possibility of online reservation. The respective time-dependent variables should either be multiplied by travel time (and the coefficient is added to the travel time coefficient), or they are omitted in the utility function (and the corresponding utility is implicitly included in the travel time coefficient by its average value).

2 To obtain VTAT we use the VTTS estimates obtained by Schmid et al. (2018), which are based on the same data source.

3 There is only one experimental attempt in the literature which introduces (technical) relationships between goods and time (Jara-Díaz et al. 2016); this model yields rather implausible results which underlines the experimental character of this branch of research. Also, the MAED data do not include expenses for external service providers, which prevents the use of another recent experimental extension: the introduction of domestic activities as a decision of households which are hiring external providers (Rosales-Salas and Jara-Díaz 2017).

4 The logarithm in Eq. (1) corresponds to the $\alpha_{k}$ parameter in Bhat's model. A difference, however, is that the logarithm has a predefined curvature, whereas $\alpha_{k}$ can be estimated.

5 In an empirical survey it can still happen that zero assignments occur, in particular, if more detailed activity classifications are used. In this case we suggest imputing reasonable values using methods available in the literature.

6 Jara-Díaz et al. (2008) used the equation as $X_{j}^{*} = \frac{{\varphi_{j} }}{{P_{j} \varPhi }}\left( {wT_{w}^{*} - E_{c} } \right)$, which is equivalent to Eq. (15) noting that $E_{j} = X_{j} P_{j}$.

7 The change in normalization was done for analytical convenience only; it does not affect the results. Please note that *α* and *β* are derived from the exponents of the Cobb–Douglas function and are therefore a-dimensional—as the original parameters.

8 'KONTIV' is the name of a travel survey design and instrument developed in the seventies by Werner Brög and associates for the German national travel diary. It has become the standard of self-administered travel surveys in German speaking countries.

9 *Φ* captures the utility associated with freely consumed goods; the remaining two input quantities in Eq. (16) are constants and do not affect the VoL: the time constraint *τ* and the parameter *Θ*, which is set to one for normalisation.

10 Another problem associated with a priori segmentation are the large confidence intervals of 'urban residents' and 'single workers'; they are a result of the low sample size of these segments (see Table 5).

11 The main explanatory variables of *T*_*W*_ are *E*_*C*_ and *T*_*C*_ [see Eq. (13)]; both show indeed a strong correlation with *T*_*W*_ in the expected direction (see Fig. 4); the wage rate *w* is no actual explanatory variable but serves to translate the money units of *E*_*C*_ into time units.

12 The respective term is $- 4E_{c} /w\left( {\tau - T_{c} } \right)\theta_{w} \left( {\varTheta + \varPhi + \theta_{w} } \right)$. All other terms in Eq. (13) respond in the opposite direction to changes of *θ*_*w*_, but these responses are smaller in size and are therefore outperformed by the former.

13 The standard deviation of *T*_*W*_ was rescaled to fit in the VoL scale of Fig. 7. The rescaled values can be perceived as 'predicted VoL' from a model with the segment-specific standard deviation of *T*_*W*_ as sole predictor: $y = - 12.36 + 239/stdev\left( {T_{W} } \right)$.

14 Each of the four main effect parameters can have an interaction term independent from the other parameters, which yields 15 possible models according to permutation rules: four models with one interaction term, six models with two interaction terms, four models with three interaction terms, and one model with four interaction terms.

15 The continuous choice models presented in this paper include the reported trips as part of the committed time and the travel cost as part of the committed expenses (see Table 3).

16 The choice probabilities are assumed to distribute logistically and were subject to a Lee-transformation in order to obtain normally distributed variables. Lee (1983) proposed a method to account for correlations in a discrete–continuous model system by transforming a-priori assumed marginal distributions for each error term into the standard normal and generating a joint multivariate normal distribution of the resulting transformed error terms. Lee’s method has been applied by many authors, e.g., Bhat (1998) and Habib (2013).
